# Supplementary material for: Effects of 4:3 Intermittent Fasting on Eating Behaviors and Appetite Hormones: A Secondary Analysis of a 12-Month Behavioral Weight Loss Intervention
Source: Nutrients. 2025 Jul 21;17(14):2385. doi: 10.3390/nu17142385 (PMC12298406; doi:10.3390/nu17142385)
Supplement: Supplementary file 1 [file nutrients-17-02385-s001.zip › nutrients-3739867-supplementary.pdf]

Table S1. Changes in eating behavior scores by randomized groups over 12 months

Variable	Month	4:3 IMF	Change from baseline in 4:3 IMF	DCR	Change from baseline in DCR	Difference in change from baseline between groups	Overall Effect p-value
BES	0	11.7 (10.4 to 13.1)		12.7 (11.4 to 14.1)			**T: < 0.05** G: 0.09 **GxT: < 0.01**
	3	10.5 (9.1 to 12.0)	**-1.2 (-2.2 to -0.1)**	11.7 (10.2 to 13.2)	-1.0 (-2.1 to 0.2)	0.2 (-1.4 to 1.7)	
	6	10.5 (9.0 to 12.1)	-1.1 (-2.4 to 0.1)	11.6 (10.0 to 13.2)	-1.1 (-2.4 to 0.2)	0.0 (-1.8 to 1.8)	
	12	9.4 (7.8 to 11.0)	**-2.3 (-3.5 to -1.1)**	12.8 (11.1 to 14.4)	0.1 (-1.3 to 1.3)	**2.4 (0.6 to 4.1)**	
Uncontrolled Eating (TFEQ – R18)	0	37.1 (33.6 to 40.6)		37.6 (34.1 to 41.2)			T: 0.16 G: 0.10 **GxT: < 0.01**
	3	33.5 (29.5 to 37.6)	**- 3.6 (-6.5 to -0.7)**	37.4 (33.3 to 41.5)	- 0.2 (-3.2 to 2.8)	3.37 (-0.8 to 7.6)	
	6	35.8 (31.7 to 39.8)	- 1.4 (-4.5 to 1.8)	39.1 (34.8 to 43.3)	1.4 (-1.9 to 4.7)	2.8 (-1.8 to 7.4)	
	12	33.0 (28.7 to 37.3)	**- 4.1 (-7.5 to -0.7)**	42.3 (37.7 to 46.9)	**4.67 (0.9 to 8.4)**	**8.8 (3.8 to 13.9)**	
Cognitive Restraint (TFEQ – R18)	0	43.5 (40.2 to 46.8)		42.5 (39.1 to 45.8)			**T: < 0.01** G: 0.58 GxT: 0.19
	3	56.4 (52.9 to 59.8)	**12.8 (9.1 to 16.6)**	60.8 (57.2 to 64.3)	**18.3 (14.5 to 22.2)**	**5.5 (0.1 to 10.9)**	
	6	55.6 (52.0 to 59.2)	**12.1 (8.5 to 15.7)**	56.2 (52.4 to 59.9)	**13.7 (10.0 to 17.5)**	1.7 (3.5 to 6.8)	
	12	54.4 (51.0 to 57.7)	**10.8 (7.5 to 14.2)**	54.8 (51.1 to 58.5)	**12.4 (8.68 to 16.0)**	1.5 (3.5 to 6.5)	
Emotional Eating (TFEQ – R18)	0	52.5 (46.3 to 58.8)		55.6 (49.2 to 61.9)			**T: < 0.01** G: 0.08 GxT: 0.37
	3	43.9 (37.9 to 49.9)	**- 8.6 (-13.8 to -3.4)**	51.9 (45.8 to 58.1)	- 3.7 (-9.0 to 1.7)	5.0 (-2.5 to 12.4)	
	6	46.8 (40.6 to 52.9)	**- 5.8 (-10.7 to -0.8)**	53.8 (47.5 to 60.2)	1.7 (-6.9 to 3.4)	4.0 (-3.1 to 11.2)	
	12	46.6 (40.4 to 52.8)	**- 5.9 (-11.1 to -0.8)**	56.3 (49.7 to 62.8)	0.7 (-4.9 to 6.3)	6.6 (-1.0 to 14.2)	
RED – 13	0	18.9 (17.1 to 20.8)		20.6 (18.8 to 22.5)			**T: < 0.01** G: 0.19 GxT: 0.27
	3	16.7 (14.6 to 18.7)	**- 2.3 (-3.8 to -0.7)**	17.3 (15.2 to 19.3)	**- 3.4 (-5.0 to -1.8)**	- 1.1 (-3.3 to 1.2)	
	6	17.3 (15.0 to 19.5)	**- 1.7 (-3.3 to 0.0)**	18.8 (16.5 to 21.1)	**- 1.8 (-3.5 to -0.1)**	-0.1 (-2.5 to 2.3)	
	12	16.2 (14.1 to 18.4)	**- 2.7 (-4.5 to -1.0)**	19.3 (17.0 to 21.7)	- 1.3 (-3.2 to 0.6)	1.42 (-3.99, 1.15)	

Results are means (95% CIs) from a linear mixed effects model with unstructured covariance using an intent-to-treat analysis; Statistically significant (P < 0.05) changes from baseline are indicated in **bold**; Abbreviations are as follows: Binge Eating Scale (BES), Three-Factor Eating Questionnaire – Revised 18-item (TFEQ-R18), Reward-based Eating Drive Scale, Revised 13-item (RED-13), 4:3 Intermittent Fasting (4:3 IMF), Daily Caloric Restriction (DCR), time effects (T), group effects (G), groups (4:3 IMF and DCR) by time (month 0 and month 12) interaction (GxT).
